# Hemoglobin Levels and Weaning Outcome of Mechanical Ventilation in Difficult-To-Wean Patients: A Retrospective Cohort Study

**DOI:** 10.1371/journal.pone.0073743

**Published:** 2013-08-28

**Authors:** Yi-Chun Lai, Sheng-Yuan Ruan, Chun-Ta Huang, Ping-Hung Kuo, Chong-Jen Yu

**Affiliations:** 1 Department of Internal Medicine, National Taiwan University Hospital, Taipei, Taiwan; 2 Department of Medicine, National Yang-Ming University Hospital and School of Medicine, Taipei, Taiwan; 3 Graduate Institute of Epidemiology and Preventive Medicine, National Taiwan University, Taipei, Taiwan; 4 Department of Traumatology, National Taiwan University Hospital, Taipei, Taiwan; University of Tübingen, Germany

## Abstract

**Introduction:**

The effect of hemoglobin levels on the weaning outcomes of mechanically ventilated patients remains under debate, particularly for the patients with difficult weaning. This study aims to evaluate the effect of hemoglobin levels on weaning outcomes in difficult-to-wean patients.

**Methods:**

This retrospective cohort study was conducted in a university-affiliated teaching hospital in Taiwan. Patients who fulfilled the criteria of difficult weaning were enrolled. Medical records were reviewed to obtain data on hemograms, biochemistry tests, transfusion records, comorbidities and weaning outcome. The association between hemoglobin levels and 30-day weaning outcomes was evaluated using a logistic regression model.

**Results:**

A total of 751 patients received mechanical ventilation during the study period, 138 of whom fulfilled the criteria of difficult weaning. Compared with the patients whose hemoglobin was <8 g/dL, those with higher hemoglobin levels were more likely to be successfully weaned (odds ratio [OR], 3.69; 95% CI, 1.22–11.15 for hemoglobin 8–10 g/dL and OR, 4.16, 95% CI, 1.30–13.29 for hemoglobin >10 g/dL). Multivariate analysis showed that the odds ratio for weaning success remained significant for hemoglobin levels of 8–10 g/dL (adjusted OR, 3.3; 95% CI, 1.07–10.15) with borderline significance for hemoglobin level > 10 g/dL (adjusted OR, 2.95, 95% CI, 0.88–9.96).

**Conclusions:**

Hemoglobin level is independently associated with weaning outcome in difficult-to-wean patients. Further studies are needed to evaluate whether a restrictive transfusion trigger for acute critical illness is also appropriate for such patients.

## Introduction

Anemia is a common problem in patients with critical illness or mechanical ventilation [1,2]. The impact of anemia on the disease outcome varies in different patient populations and disease status. Mild anemia seldom requires transfusion therapy, however severe anemia cases may warrant transfusion of red blood cells (RBC) to correct unfavorable physiological effects. Previous studies have suggested different transfusion triggers of RBC for different clinical settings [3]. For example, current evidence and practice guidelines suggest a restrictive strategy with a transfusion trigger of hemoglobin level <7 g/dL for critically ill patients [4,5]. The rationale against a liberal transfusion strategy is that transfusion-related complications outweigh the advantages of correcting anemia. Cumulative data show that a transfusion may increase the risk of acute transfusion reaction and subsequent nosocomial infection by the immunosuppressive effects of allogeneic transfusion [3,6,7]. Therefore, a transfusion trigger is a tolerable threshold of anemia compromised by the adverse effects from transfusion but is not a truly appropriate hemoglobin level for that patient. Thus the impact of hemoglobin levels on the outcome of a specific patient group should be studied beyond the scope of a transfusion trigger because the risk of transfusion-related adverse effects is partly preventable [8,9].

The role of hemoglobin levels on the weaning outcomes of mechanically ventilated patients is still under debate [10–13], and the data are especially limited for patients with difficult weaning. Difficult-to-wean patients are more likely to benefit from higher hemoglobin levels compared to those at an early stage of respiratory failure because adequate oxygen delivery mediated by sufficient hemoglobin is crucial during the weaning process [14,15]. It is known that lower hemoglobin levels decrease arterial oxygen content, compromise oxygen delivery and increase respiratory muscle load during the weaning process [14,15].

There are no conclusive recommendations regarding a suitable transfusion trigger in the current guidelines on ventilator weaning because of the inconsistent results in various studies [16,17]. Two case series showed that the higher hemoglobin levels achieved after a transfusion were associated with a reduction in the work of breathing and increased weaning success in patients with chronic obstructive pulmonary disease (COPD) [11,12]. However, subgroup analysis in a randomized study, the Transfusion Requirements in Critical Care (TRICC) trial, showed that a liberal transfusion strategy failed to decrease the duration of mechanical ventilation [10]. This discrepancy resulted from different study populations and targeted hemoglobin levels, and more studies are needed to clarify the disparity between different patient groups. To test the hypothesis that a lower hemoglobin level is associated with worse weaning outcomes in difficult-to-wean patients, we conducted a retrospective cohort study to evaluate the effects of hemoglobin levels on the weaning outcomes of patients with mechanical ventilation.

## Materials and Methods

### Study population

This retrospective cohort study was conducted at the National Taiwan University Hospital Yunlin Branch, a university-affiliated teaching hospital in Taiwan. A protocol driven strategy is used for ventilator weaning in the hospital, the details of which have been described in a previous study [18]. A ventilator registry database was used to identify all patients receiving mechanical ventilation for more than 24 hours from April 2008 to April 2009. The study cohort of difficult-to-wean patients in this study included patients with difficult weaning and prolonged weaning defined in a previous consensus statement [16]. The patients identified as being difficult weaning included those with prolonged weaning, as they would first meet the criteria of difficult weaning before those of prolonged weaning. Therefore we used the definition of difficult weaning as the inclusion criteria in this study: (1) patients who failed spontaneous breathing trials (SBT) two or more times; or (2) patients who required as many as 7 days from the first SBT to successful weaning. The exclusion criteria included patients with simple weaning, death before attempted weaning, and incomplete follow-up due to transfer. Medical records were reviewed to obtain data on hemograms, biochemistry tests, simplified acute physiology score II (SAPS II) and comorbidities. The frequency and dosage of RBC transfusions were also recorded. The decision on transfusion strategy is made by the physician. This study was approved by the Research Ethics Committee of the National Taiwan University Hospital (Approval number: 200802032R) and the need for informed consent was waived.

### Exposure and Outcomes

Hemoglobin levels were stratified into three strata by cut-off values of 8 and 10 g/dL in this study (< 8 g/dL, 8-10 g/dL and > 10 g/dL), and these three strata represented three exposure levels. The cut-off values were determined according to practice guidelines in which a hemoglobin level of 8-10 g/dL was suggested to be appropriate for ventilator weaning [17]. The impact of hemoglobin levels on weaning outcomes was assumed to be an acute effect, and the hemoglobin levels in the acute stage of respiratory failure before attempted weaning were assumed to have little impact on weaning outcomes. The hemoglobin level at the end of the weaning period or follow-up was chosen as the exposure level for the patient. The follow-up period for each patient was 30 days from the initiation of mechanical ventilation. The primary endpoint of the study was successful weaning in 30 days, and successful weaning was defined as liberation from mechanical ventilation for more than 48 hours. Patients who died before successful weaning were considered as weaning failure.

### Statistical Analysis

The association between hemoglobin levels and 30-day weaning outcomes was tested using logistic regression and odds ratios (OR) and 95% confidence intervals (95% CI) were calculated. Variables with a p value of <0.2 in the univariate regression were selected to determine the final model. Model selection by stepwise method was also performed. The three categories of hemoglobin levels were fit as a continuous variable to assess trends in odds ratios. The time-to-event curves for weaning success were generated using the Kaplan-Meier method. SAS statistical software (SAS, Version 9.2) was used for data analysis. All tests were two-sided and *P* values of <0.05 were deemed to be significant.

## Results

**Figure 1:**
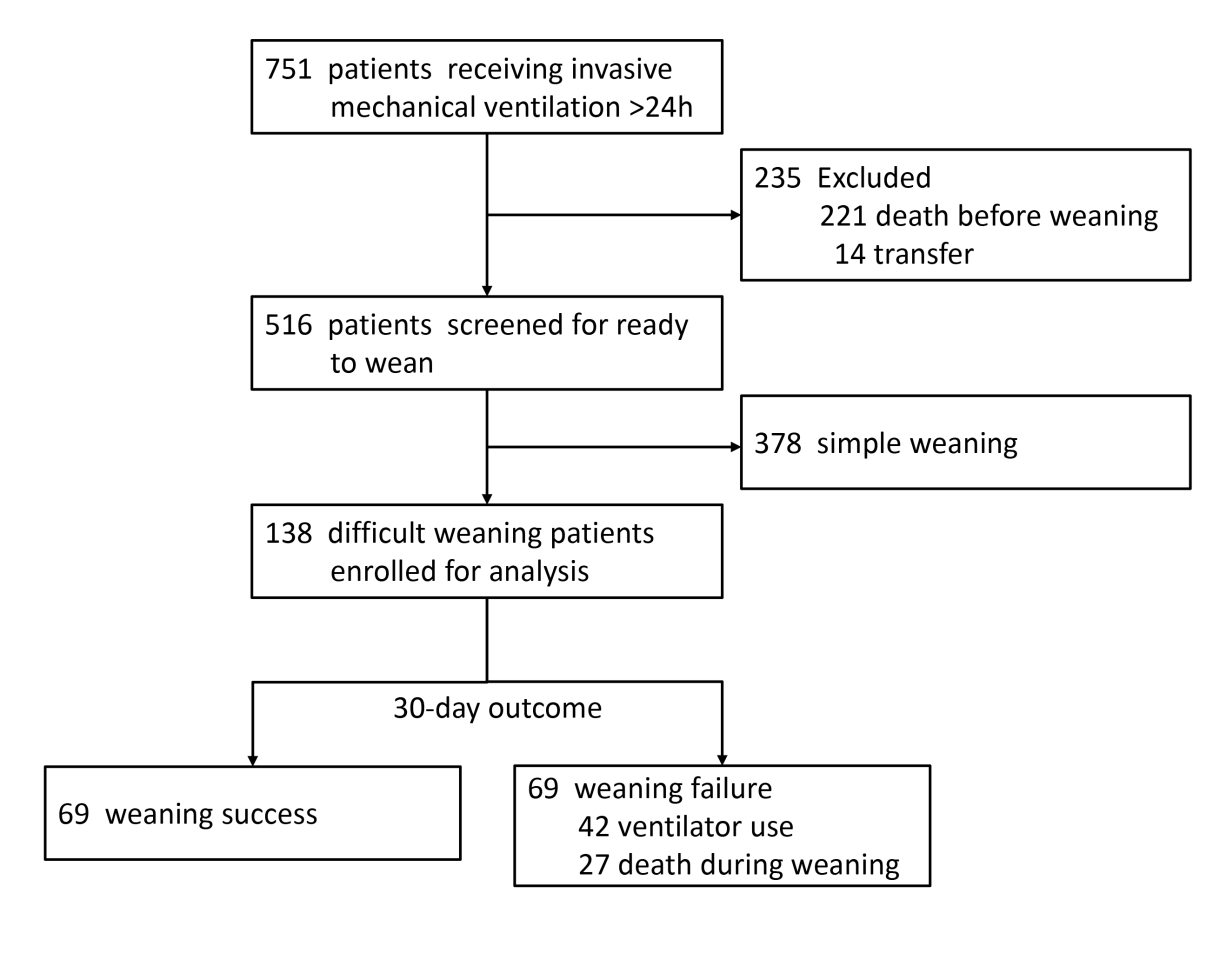
Study flow.

A total of 751 patients receiving invasive mechanical ventilation for more than 24 hours were screened for eligibility. Among them, 138 patients who fulfilled the criteria of difficult or prolonged weaning were included for analysis. Figure 1 shows the study flow, and the characteristics of the study cohort are summarized in Table 1. The mean hemoglobin level at the initiation of mechanical ventilation was 10.7 g/dL and the average interval in blood testing for complete blood count was 2.5 ± 1.8 days.

**Table 1:** Characteristics of the study population.

**Characteristics**	**N (%) or Mean (SD)**
Age, yr	73 (13)
Female sex	57 (41)
Body mass index	21.2 (4.5)
Reason of ventilator use	
Medical	120 (87)
Postoperative	18 (13)
SAPS II score at ventilator start	59 (15)
SAPS II score at the 1^st^ SBT	46 (14)
PaO_2_/FiO_2_ ratio at ventilator initiation	263 (119)
Hemoglobin level, g/dL	
Ventilator start	10.7 (2.2)
Ventilator day 30	9.5 (1.4)
White blood cell count, 10^9^ cells/L	13.9 (7.1)
Platelet count, 10^9^/L	218 (107)
Creatinine level, mg/dL	1.7 (1.4)
Transfusion with red blood cells	97 (70)
Comorbidity	
Renal insufficiency, CCR<30 mL/min	52 (38)
Diabetes	45 (33)
Gastrointestinal bleeding	28 (20)
Malignancy	25 (18)
Coronary arterial disease	19 (14)
COPD	15 (11)

CCR = creatinine clearance rate; SBT = spontaneous breathing trial

N=138

**Figure 2:**
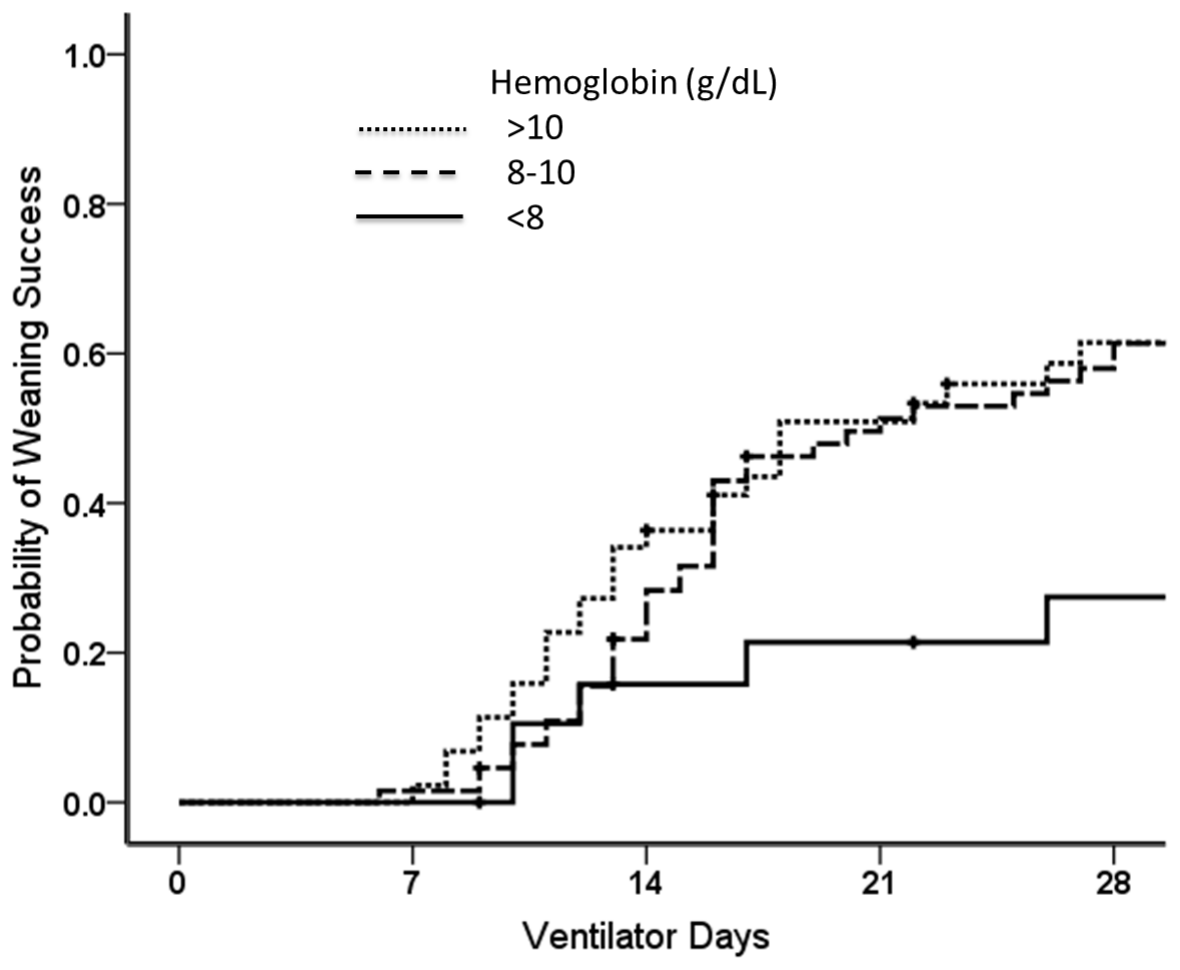
Probability of weaning success in patients with different hemoglobin levels (*P* = 0.06 by the log-rank test).

Table 2 shows the odds ratios for weaning success with regards to hemoglobin levels, age, sex and comorbidity. In univariate analysis, hemoglobin levels of 8-10 g/dL and > 10 g/dL were positively associated with weaning success compared to a hemoglobin level of < 8 g/dL (OR, 3.69; 95% CI, 1.22 to 11.15 and OR, 4.16, 95% CI, 1.30 to 13.29, respectively). In addition, there was a significant dose–response relationship in hemoglobin levels (*P* for trend= 0.03). Figure 2 demonstrates the curves of probability of weaning success for patients with different hemoglobin levels.

**Table 2:** Odds ratio for weaning success in 30 days.

Variables	Weaning outcome		Unadjusted OR (95%CI)	P value	Adjusted OR (95%CI)	P value
	Success N=69	Failure N=69				
Hemoglobin level, g/dL						
<8	5	16	1.00			
8-10	38	33	3.69 (1.22-11.15)	0.02	3.30 (1.07-10.15)	0.04
>10	26	20	4.16 (1.30-13.29)	0.02	2.95 (0.88-9.96)	0.08
P_trend_				0.03		
Age, yr						
<65	18	18	1.00			
65-75	14	24	0.58 (0.23-1.48)	0.26		
>75	37	27	1.37 (0.60-3.11)	0.45		
Sex						
female	31	26	1.00			
male	38	43	0.74 (0.38-1.46)	0.39		
RBC transfusion (Unit/week)	0.7±1.0	0.8±1.1	0.94 (0.68-1.30)	0.71		
SAPS II, ventilator start	60±15	59±15	1.00 (0.98-1.02)	0.82		
SAPS II, 1^st^ SBT	42±12	49±16	0.97 (0.95-0.99)	0.02	0.97 (0.95-1.00)	0.05
PaO_2_/FiO_2_ ratio	262±116	264±123	1.00 (0.99-1.00)	0.92		
Comorbidity						
Malignancy	12	13	0.91 (0.38-2.16)	0.83		
Diabetes	22	23	0.94 (0.46-1.91)	0.86		
Renal insufficiency	25	27	0.88 (0.44-1.76)	0.73		
Gl bleeding	12	16	0.70 (0.30-1.61)	0.40		
COPD	7	8	0.86 (0.29-2.52)	0.79		
CAD	8	11	0.69 (0.26-1.84)	0.46		

CAD = coronary artery disease; COPD = chronic obstructive pulmonary disease; RBC = red blood cells; SAPS = simplified acute physiology score; SBT = spontaneous breathing trial

There were total 97 patients (69%) that received RBC transfusion during study period. The numbers of patients receiving RBC transfusion in each subgroup are summarized in Table 3. There was no significant difference in the proportion of patients with different hemoglobin levels receiving RBC transfusion both in weaning success and weaning failure groups. Among patients with a hemoglobin level of >10 g/dL, RBC transfusion was significantly associated with weaning failure.

**Table 3:** Number of patients receiving red blood cells transfusion.

Hemoglobin	Weaning success			Weaning failure		P value
	Transfusion N=40	No transfusion N=29		Transfusion N=57	No transfusion N=12	
<8 (N = 21)	3	2		15	1	0.13
8-10 (N = 71)	26	12		25	8	0.49
>10 (N = 46)	11	15		17	3	0.003
	P = 0.11			P = 0.28		

Multivariate analysis showed that the odds ratio for weaning success remained significant for a hemoglobin level of 8-10 g/dL (adjusted OR, 3.3; 95% CI, 1.07-10.15) and borderline significance for a hemoglobin level of > 10 g/dL (adjusted OR, 2.95, 95% CI, 0.88-9.96). The SAPS II score was negatively associated with weaning success (adjusted OR, 0.97, 95% CI, 0.95-1.00). The results of model selected by a stepwise method showed consistent findings. By stepwise method, only hemoglobin levels, age and SAPS II score left in the final model. The adjusted ORs for hemoglobin level of 8-10 g/dL and > 10 g/dL were 3.46 (95% CI, 1.11-10.85) and 2.77 (95% CI, 0.80-9.54) respectively. In addition, multivariate analysis by controlling disease severity (SPAS II score) and comorbidities were also performed and the results were similar to the presented analysis (data not shown).

## Discussion

The findings of this study suggest that hemoglobin levels are associated with weaning outcomes in difficult-to-wean patients. The unadjusted results found a dose–response relationship between the three hemoglobin levels (< 8 g/dL, 8-10 g/dL and > 10 g/dL) and weaning success. After controlling for comorbidity and disease severity, the adjusted odds ratio for weaning success remained significant for a hemoglobin level of 8-10 g/dL and borderline significance for a hemoglobin level of > 10 g/dL. Current evidence suggests a restrictive transfusion trigger of 7 g/dL for acute critically ill patients [4]. However, it is not clear whether this rule can be generalized to difficult-to-wean patients. For the patients with poor cardiopulmonary reserve, the benefits of increasing the hemoglobin level may outweigh the risk of transfusion because they are more susceptible to the decline of oxygen delivery caused by low hemoglobin levels. Further clinical trials are needed to explore an optimal transfusion trigger for this patient population. Our study only aimed to evaluate the association between hemoglobin levels and weaning outcome but was unable to determine a transfusion trigger due to the retrospective nature of the study design.

The crude and adjusted odds ratios for weaning success were similar for a hemoglobin level of 8-10 g/dL but the adjusted odds ratio was smaller than crude odds ratio for a hemoglobin level of >10 g/dL (Table 2). The crude odds ratio represented a measure of the overall effect of hemoglobin level on weaning outcome, including the direct effect of anemia itself and the indirect effect of comorbidity and disease severity associated with anemia. Therefore, the advantage of a better underlying condition associated with a higher hemoglobin level would be corrected after adjusting for comorbidity and disease severity. This may explain why a higher hemoglobin level of > 10 g/dL did not confer an additional advantage to those with a level of 8-10 g/dL in multivariate analysis. Disease severity was shown to be an important factor affecting weaning outcome [19]. In this study, SAPS II score was also an independent factor for weaning outcome. In addition, disease severity also affects hemoglobin level of a patient. However, the effect of hemoglobin level on weaning outcome remained significant after adjusted SAPS II score in multivariate analysis.

The proportion of patients with different hemoglobin levels receiving RBC transfusion did not differ significantly in both weaning success and weaning failure groups. This suggests that the different hemoglobin levels in this study population might not be explained by the effect of transfusion but reflected underlying medical conditions. However, confounding by indication is a concern because physicians are more likely to maintain higher hemoglobin levels for patients with more severe comorbidities. The effect of transfusion on weaning outcome had better been evaluated by experimental studies. Our study only aimed to evaluate the association between hemoglobin levels and weaning outcome.

Determining the transfusion trigger for difficult-to-wean patients is an area of clinical uncertainty and practice variation. Although our study findings showed a significant association between hemoglobin levels and weaning success, this cannot be simply translated to a transfusion trigger for difficult-to-wean patients. A transfusion trigger indicates a threshold at which the benefit of increasing hemoglobin level by transfusion exceeds the risk of transfusion-related adverse effects. Our study only evaluated the effects of hemoglobin levels on weaning outcomes but not the harm related to the transfusion due to the inherent limitation of its observational design. Clinical trials are needed to identify the appropriate transfusion trigger for this specific population. In addition, because hemoglobin levels independently affect weaning outcomes, several non-transfusion strategies may be used to increase hemoglobin levels, such as nutritional support, screening gastrointestinal or other blood loss, and iron preparations or erythropoietin agonists in certain clinical situations.

The physiological basis of the impact of anemia on weaning outcome is multifaceted. Hemoglobin plays a critical role in oxygen delivery and may affect cardiac workload, work of breathing and respiratory muscle endurance [12,14,15]. Several observational studies have provided supportive evidence for these findings [11,12,20]. In a prospective cohort study, a hemoglobin level of less than 10 g/dL was associated with extubation failure [20]. Schonhofer and colleagues reported that RBC transfusions decreased work of breathing and facilitated weaning from mechanical ventilation [11,12]. Practice guidelines on ventilator weaning have also considered anemia to be an important issue in dealing with weaning failure [16,17].

Hébert and colleagues reported important data by subgroup analysis of a large randomized trial (TRICC). The post-hoc analysis found that a liberal transfusion strategy did not improve weaning outcomes but was rather associated with a trend of worse weaning outcomes [10]. There are two major disparities between the TRICC trial and the current study in terms of patient population and comparison levels of hemoglobin. The TRICC trial evaluated all mechanically ventilated patients, however the current study focused on difficult-to-wean patients. In addition, the current study showed that a hemoglobin level of 8-10 g/dL was associated with better weaning outcomes than a hemoglobin level of less than 8 g/dL. In the TRICC trial, the comparison group included a hemoglobin level of up to 12 g/dL, which may exceed the baseline hemoglobin level of some chronically ill patients. In the current study, the mean hemoglobin level at the initiation of mechanical ventilation was only 10.7 g/dL. Using transfusions to maintain a supra-baseline hemoglobin level may result in frequent RBC transfusions and subsequently cause harm.

Our results should be interpreted with caution because of several limitations that are worth noting. First, as there is no universal protocol for transfusion practice and due to the retrospective nature of the study design, the mechanism behind the transfusion and residual confounding factors may have affected the results; however we did deal with the confounding of transfusion and comorbidity by utilizing multivariate regression analysis. Second, the frequency of blood testing varied, and there may have been information bias caused by disease severity. However, the data sampling density of hemoglobin level was high, suggesting that this bias was very limited.

## Conclusions

The findings of this observational study suggested that hemoglobin level was an independent factor associated with weaning outcomes in difficult-to-wean patients. This raises the question as to whether a restrictive transfusion trigger of 7 g/dL for acute critically ill patients is also appropriate for difficult-to-wean patients. Further clinical trials are needed to explore an optimal transfusion trigger for such patients.
